# The formation of intestinal organoids in a hanging drop culture

**DOI:** 10.1007/s10616-018-0194-8

**Published:** 2018-01-25

**Authors:** Malgorzata Panek, Maja Grabacka, Malgorzata Pierzchalska

**Affiliations:** Department of Food Biotechnology, Faculty of Food Technology, The University of Agriculture in Kraków, Balicka 122, 30-149 Kraków, Poland

**Keywords:** Chicken epithelial cells, Spheroids, Organoids, Three-dimensional cell culture, Enterocytes, Intestine

## Abstract

Recently organoids have become widely used in vitro models of many tissue and organs. These type of structures, originated from embryonic or adult mammalian intestines, are called “mini guts”. They organize spontaneously when intestinal crypts or stem cells are embedded in the extracellular matrix proteins preparation scaffold (Matrigel). This approach has some disadvantages, as Matrigel is undefined (the concentrations of growth factors and other biologically active components in it may vary from batch to batch), difficult to handle and expensive. Here we show that the organoids derived from chicken embryo intestine are formed in a hanging drop without embedding, providing an attractive alternative for currently used protocols. Using this technique we obtained compact structures composed of contiguous organoids, which were generally similar to chicken organoids cultured in Matrigel in terms of morphology and expression of intestinal epithelial markers. Due to the simplicity, high reproducibility and throughput capacity of hanging drop technique our model may be applied in various studies concerning the gut biology.

## Introduction

The intestinal epithelium is one of the largest (in terms of the surface) tissue of the body with a particularly high renewal rate. In the intestine of all adult vertebrates, the mature enterocytes and the other differentiated epithelial cells originate from mitotically active intestinal stem cells located at the base of the crypts. In avian embryos and in newly hatched chicks the situation is more complicated, as proliferating cells are detected also outside the crypt region, which is not fully developed (Uni et al. 1998). Nevertheless, the self-renewing potential of avian intestinal stem cells allows the proper intestine function throughout life. In both mammals and birds the role of the intestinal epithelium is not restricted to nutrient absorption and to the formation of a protective barrier between the internal and external environment. Intestinal mucosa also actively regulates the host-parasite and host-microbiota interactions, being involved in the induction of innate or adaptive immune responses (Chopra et al. 2010). It is also the main place of action for biologically active food ingredients, before they are absorbed into the bloodstream. These rationales justify the search for in vitro models of the mammalian gut, which might be relevant not only for physiology and cell biology, but also for food science studies (Beyaz et al. 2016), veterinary (Khalil et al. 2016), cancer research (Janeckova et al. 2015) and gastroenterology (Mohamed et al. 2015).

Due to profound and growing role of modern poultry industry in food production worldwide, the avian gut physiology and immunity are also considered an important issue both from scientific and economical point of view. Notably, the successful development and introduction of in ovo injection systems as a route to apply various biologically active substances open new possibilities to improve the post-hatch chicken performance by supporting immunity and gut maturation (Roto et al. 2016). This idea creates the need for in vitro cell culture models originated from late embryonic avian gut that could be used to evaluate the influence of various compounds and microorganisms on intestinal epithelium maturation and function or intestinal immunity.

The primary culture of the normal intestinal epithelial cells is relatively difficult because of heterogeneity and polarity of epithelial cells, susceptibility to anoikis (a type of apoptosis caused by loss of cellular contacts) and a profound role of close cell–cell and cell–extracellular matrix (ECM) interactions in keeping their proliferation potential. Currently, the three dimensional (3D) culture of the intestinal organoids serves as a model for gut physiology studies (Huch and Koo 2015). The major advantage of 3D in vitro models involves their ability to partially reconstruct the dynamics typical for the tissue, and therefore providing researchers with a more physiological context for their experiments. To establish the 3D conditions, cells can be embedded in ECM proteins, cultured as aggregates or placed on the artificial 3D scaffolds and matrices (Fennema et al. 2013; Breslin and O’Driscoll 2013). At the same time, the method of cell culture in “hanging drop” has regained a considerable interest. The technique, developed by cell culture pioneers in early twentieth century, physically favors cell-to-cell interactions due to the lack of rigid support (glass or plastic surface) or solidified ECM scaffold. Spheroids are self-organizing cell aggregates, which are formed when the cell–cell interactions substitute the cell–substratum contact (Foty 2011). Hanging drop culture principle was used to establish spheroids from various types of cells and tissues, like embryoid bodies (Wang and Yang 2008), normal microtissues (Beauchmp et al. 2015; Kelm et al. 2004) and tumors (Kelm et al. 2003) with some technical variations. Historically, a hanging drop technique was developed by Harrison (1907) and used to culture nerve fibres. Later, the method was adopted by others to obtain the multicellular tumor spheroids (Kelm et al. 2003). Kelm et al. put small volume of cell suspension into the wells of a special microtiter plate. They took advantage of the fact that surface tension keeps a hanging drop of liquid in place, while gravity force causes the concentration of cells at the drop’s bottom. The hanging drop technique allows obtaining in relatively simple way the large numbers of uniform spheroids with approximately 100% efficiency—one spheroid per one drop. The tightly packed spheroids are formed more frequently than the loose cell aggregates, but the morphology of spheroids depends on the cell type used (Breslin and O’Driscoll 2013). Numerous advantages of this technique include the limited usage of ECM and no need of artificial scaffold. The establishment of hanging drop cultures has become even more convenient since the development of two new special platform plates (e.g. produced by 3D Biomatrix or InSphero), which increase throughput of this method and overcome the problems with the exchange of media.

So far, the hanging drop technique has not been used to culture organoids originated from intestinal epithelium. Here we present a novel in vitro model based on the hanging drop culture of organoids created by cells isolated from embryonic chicken intestine.

## Methods

### Tissue isolation and organoid culture

Epithelial tissue was obtained from the small intestines of 18-day old chicken embryos as previously described (Pierzchalska et al. 2016). The cell pellet was re-suspended in the DMEM/F12 (PAN-Biotech GmbH, Aidenbach, Germany) culture medium supplemented with BD Matrigel™ (5%, BD Biosciences, San Diego, CA, USA), antibiotic–antimycotic solution (Zell Shield, Minerva Biolabs, Berlin, Germany), Insulin–Transferrin–Selenium Premix (BD Biosciences, San Jose, CA, USA), Epidermal Growth Factor (20 ng/ml, PeproTech, London, UK), prostaglandin E_2_ (5 µg/ml, Cayman, Ann Arbor, MI, USA) and WNT3a (10 ng/ml, R&D Systems, Minneapolis, MN, USA). The small aliquots of cell suspension (40 µl per well) were introduced into the 96-well Perfecta3D Hanging Drop Plate (3D Biomatrix, Ann Arbor MI, USA) and kept at 37 °C, 5% CO_2_. Every second day 4 µl of medium was aspirated and a portion of fresh medium (8 µl) was added. Simultaneously, the culture of chicken epithelial organoids in Matrigel was conducted in accordance with previously established protocol (Pierzchalska et al. 2012) in cell culture inserts.

### Immunoblotting

The spheroids (pooled from eight wells) were transferred to an eppendorf tube by blowing air from a 200 µl pipette through the holes in the plate, and subsequently centrifuged (1000×*g*, 5 min). After removal of supernatants, pellets were lysed with RIPA buffer (Thermo Fisher Scientific, Waltham, MA, USA) containing cocktail of protease inhibitors (cOmplete™, Roche Diagnostic, Mannheim, Germany). Total protein was also isolated from spheroids cultured in Matrigel as previously described (Pierzchalska et al. 2012). Protein concentration was evaluated using Pierce™ BCA Protein Assay Kit (Thermo Fisher Scientific, USA). The SDS-PAGE electrophoresis of 20 µg of the whole cell protein extracts was performed in 4–20% gradient gel (Precise™ Tris–Glycine Gel, Thermo Fisher Scientific) followed by protein transfer to the nitrocellulose membranes. Membranes were incubated in 5% bovine serum albumin in PBS-Tween (0.05%) for 1 h and then overnight at 4 °C with monoclonal antibody against leucine rich repeat containing G protein-coupled receptor 5, Lgr5 (1:1000 C16, Santa Cruz Biotechnology, Santa Cruz, CA, USA; a stem cell marker) or in 5% free-fat milk in PBS-Tween (0.05%) for 1 h and then overnight at 4 °C with monoclonal antibody against: chicken Villin (1:2000, MCA292, AbD Serotec, Raleigh, NC, USA; an enterocyte marker); Sucrose-isomaltase (1:1000, SAB102141, Sigma Aldrich, St. Louis, MO, USA; an enterocyte brush border marker); Sox9 (1:200, H90, Santa Cruz Biotechnology; an intestinal progenitor/stem cell marker); proliferating cell nuclear antigen, Pcna (1:3000, P8825, Sigma Aldrich; a proliferating cell marker); alpha-smooth muscle actin, α-SMA (1:2000, 1A4, Sigma-Aldrich; a myofibroblast marker). Growth factor receptor-bound protein 2 (Grb2) or β-actin (Actin) were reference proteins and their immunodetection was performed using mouse anti-Grb2 monoclonal antibody diluted 1:3000 (610112, BD Bioscience) or anti-β-actin monoclonal antibody diluted 1:5000 (A2228, Sigma-Aldrich) on the same membranes. After 12 h the membranes were washed three times in PBS-Tween and incubated for 1 h (in room temperature) in the HRP conjugated anti-mouse IgG or anti-rabbit IgG secondary antibody (Cell Signaling Technology Inc., Danvers, MA, USA) diluted 1:5000 in 5% fat free milk in PBS-Tween or in the HRP conjugated anti-goat IgG secondary antibody (Santa Cruz Biotechnology), diluted 1:4000 in 5% bovine serum albumin in PBS-Tween and washed three times. The ECL Plus Reagent (Cell Signaling Technology Inc.) was used to visualize bands.

### Histochemistry, detection of proliferating or dead cells and measurement of organoid size

“Hanging” spheroids were transferred to Eppendorf tubes with the pipette using an air pressure, as described above. If necessary, the spheroid suspension was centrifuged (700×*g*, 5 min). The supernatants were discarded and the spheroids were fixed by re-suspension in 3.7% buffered formaldehyde. Next, they were stained with Hoechst 33342 (5 µg/ml, Thermo Fisher Scientific) or propidium iodide (1 µg/ml, Thermo Fisher Scientific), transferred into a glass bottom plate (12-well, Nb.0 Coverslip, 10 mm glass diameter, uncoated, MatTek Corporation, Homer Ave, Ashland, MA, USA) and subjected to the microscopic observation and analysis in the AxioObserver Z.1 microscope with Zen 2012 software (Zeiss, Oberkochen, Germany). Click-iT^®^ EdU Imaging Kit (Thermo Fisher Scientific) was used to detect cells in S-phase of cell cycle. The organoids cultured in hanging drop were incubated in medium with thymidine analog EdU (40 µM) for 12 h, then transferred to Eppendorf tubes, centrifuged (1000×*g*, 5 min) and stained according to the manufacturer’s protocol. The area of organoids projections was measured in images taken on the second day of culture after manually contouring their shapes using Zen 2012 software (Zeiss) and the number of proliferating cells was counted. The mean area value was calculated for each culture condition tested. Statistical analysis was performed using the planned contrast method, in the Statistica 10 software.

Alternatively, the fixed “hanging” organoids were preserved in the HistoGel™ blocks (Thermo Fisher Scientific) and embedded in paraffin. Also the Matrigel layer containing spheroids was fixed in 3.7% buffered formaldehyde and embedded in paraffin. Subsequently, obtained paraffin blocks were processed according to a standard histological protocol. Thin sections obtained from paraffin blocks were deparaffinized and stained by standard hematoxylin/eosin method.

### RNA isolation and RT-PCR

The total RNA isolation was performed using TRI Reagent (Sigma-Aldrich) according to the manufacturer’s instruction. Transcriptor Reverse Transcriptase kit (A&A Biotechnology, Gdańsk, Poland) with oligo(dT) primers was used to transcribe 2 μg RNA into cDNA. Next, PCR reaction was carried out with specific primers for *Gallus gallus* genes: *villin* (forward 5′-CGGCCCACCTGATGGCGATC-3′, reverse 5′-GCTGGTCTTGCCGCCCAGAG-3′), *sox9* (forward 5′-CCCCAACGCCATCTTCAA-3′, reverse 5′-CTGCTGATGCCGTAGGTA-3′), *cdxA* (forward 5′-TAGGTTGCCCAGAGGGGCCG-3′, reverse 5′-CTCCTGTGTCCCAGCACGCC-3′), *cdxB* (forward 5′-AACAAGTTCCCTGTTCCCACCAC-3′, reverse 5′-GCAGCAGCACGAACTC CCTGA-3′) and *β-actin* (forward 5′-CATTGCCCCACCCTGAGCGCA-3′, reverse 5′-TGGTACCGGCTCCTCCCAGC-3′).

## Results and discussion

**Fig. 1:**
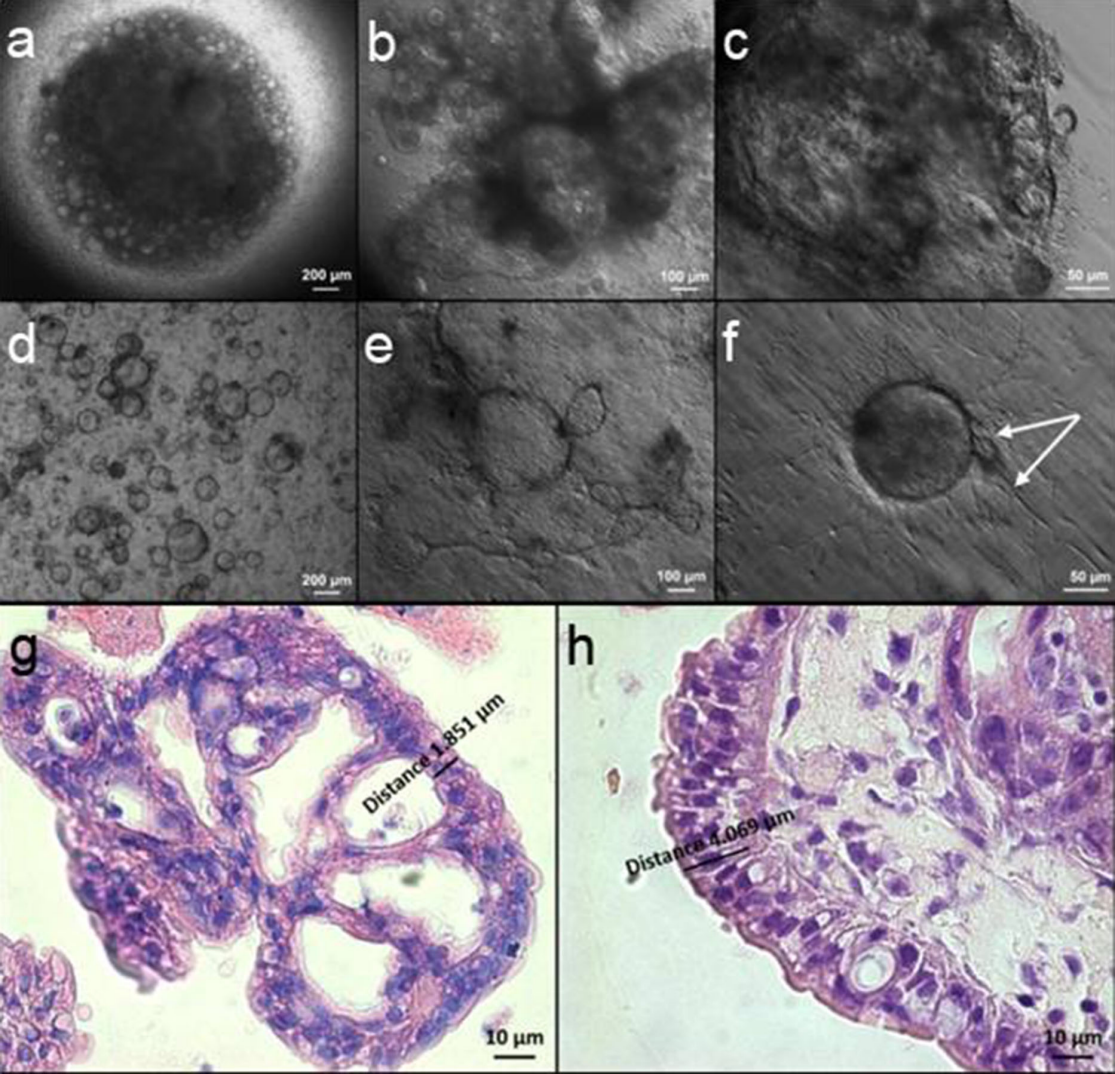
The comparison of epithelial organoids cultured in hanging drop and in Matrigel. Epithelial organoids were cultured for 4 days in hanging drop (**a**–**c**, **h**) or in Matrigel (**d**–**g**). Cultures were observed under inverted microscope equipped with differential interference optics (**a**–**f**) or embedded in paraffin, sectioned and stained with haematoxylin–eosin and observed with bright field microscopy (**g**, **h**). The white arrow on **f** shows fibroblasts expanding from the spheroids into Matrigel. Note that fibroblasts are rather not present in organoids formed without contact with solid support (**c**). Epithelial cells in organoids formed in hanging drops are more elongated in comparison to epithelial cells covering Matrigel-embedded organoids (compare **h**, **g**). (Color figure online)

**Fig. 2:**
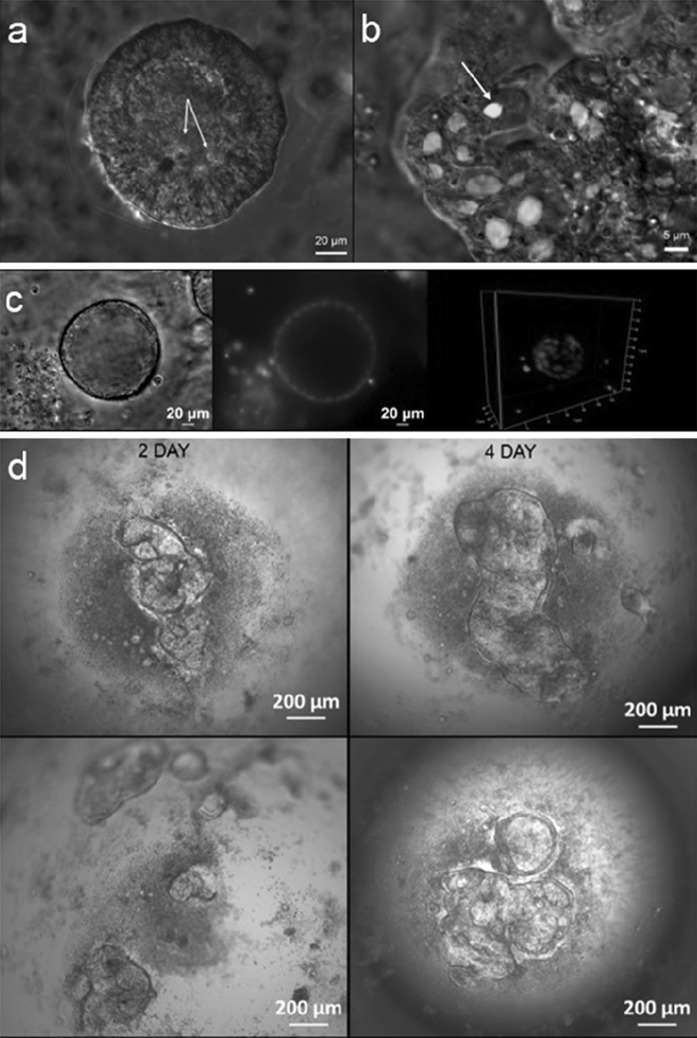
Features of organoids cultured in hanging drop. **a** Live intestinal organoids from 4-day-old cultures were stained with propidium iodide (1 µg/ml) to visualize dead cells and cells debris. The image shows that organoid wall is consisted of tightly adjacent epithelial cells, white arrows indicate some death cells present in the lumen only. **b** Alternatively, fixed organoids were stained with Click-iT^®^ EdU Imaging Kit to detect proliferative cells, white arrow shows a cell in S-phase of cell cycle which is situated in organoid wall (green color), all nucleus of cells were stained with Hoechst 33342 (blue color). **c** Fixed organoids cultures were stained with Hoechst 33342 (5 µg/ml) and photographed with an inverted fluorescence microscope with ApoTome attachment (to create 3D reconstruction, images were collected from 20 different planes). **d** The growth of organoids in two separated hanging drop cultures were followed and photographed on day 2 and 4 from plating. Notice the changes in appearance of the same culture by comparing the images in left and right columns in the same row. (Color figure online)

We show here that intestinal epithelial organoids can be effectively created in a hanging drop condition, if only the medium is supplemented with low amount of Matrigel. In our model, the cells aggregate at the drop bottom and form a consistent structure comprised of joined organoids in medium containing 5% of Matrigel (Figs. 1a, b, 2d). When cultured inside the gel composed of 50% Matrigel individual organoids are not in such a close contact (Fig. 1d, e). Usually, a single organoid in a hanging drop has the shape of empty sphere and its wall is composed of the strictly contiguous epithelial cells (Fig. 2c). Within some organoids dead cells or cell debris are detected (Fig. 2a). The obtained cultures are quite dynamic—individual organoids are growing, can fuse with each other and, as a consequence, the whole appearance of the compact structure changes in the onset of culture (Fig. 2d). A small but clearly visible fraction of proliferating cells was observed in the “hanging” organoids (Fig. 2b). The intestinal chicken organoids from hanging drop culture presented a similar morphology to intestinal organoids, derived from tissue fragments immersed in Matrigel (Pierzchalska et al. 2012), but there were also some noticeable differences between these two types of culture. The analysis of histological sections revealed that the epithelial cells of organoids cultured in hanging drop are more elongated in comparison to those building walls of Matrigel embedded organoids (compare Fig. 1g, h). The dynamically evolving morphology of organoids in hanging drop most likely results from closer contact between individual organoids (the formation of the compact structure composed of contiguous organoids due to gravity force). We showed that the culture of intestinal epithelial organoids is possible and effective without the solid ECM support. We assume that the lack of gelified medium may also contribute to the observed greater flexibility of organoids in hanging drop culture.

**Fig. 3:**
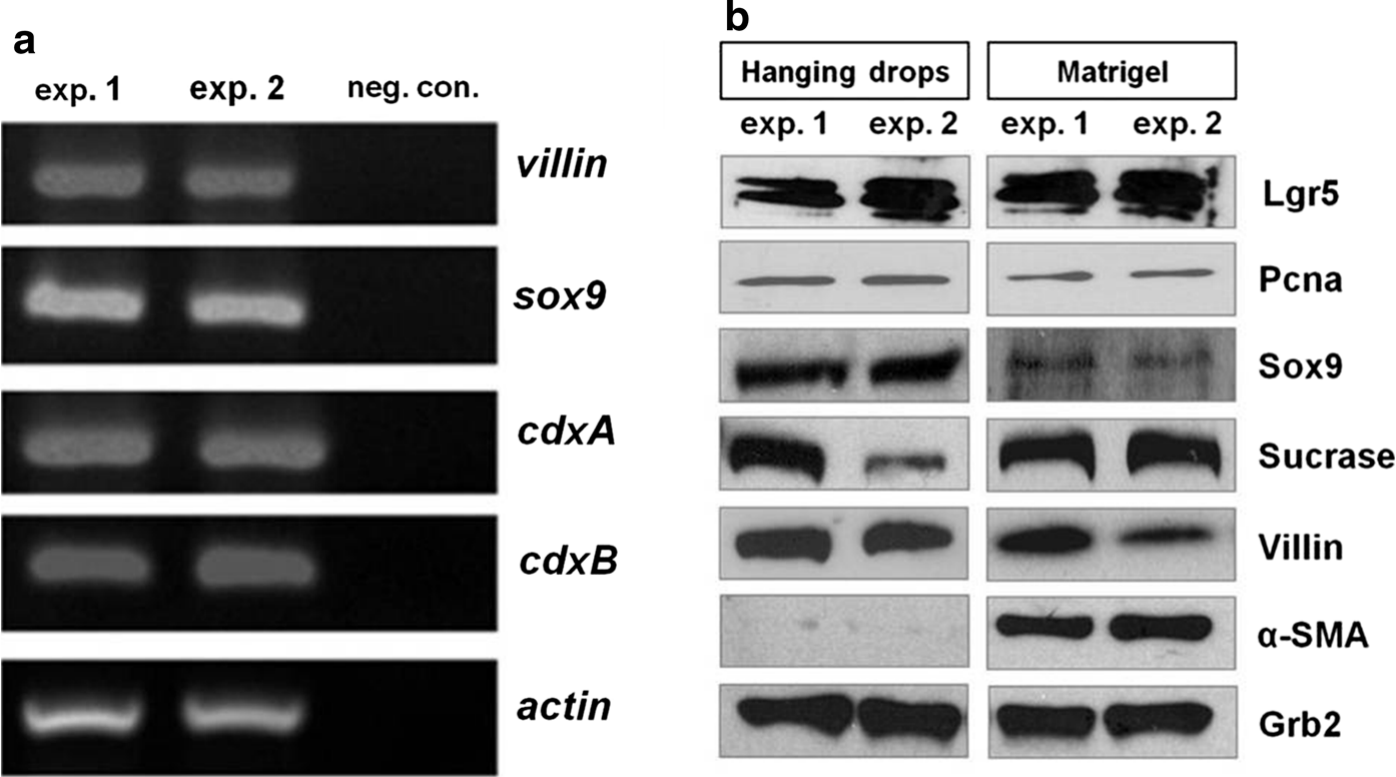
Detection of markers characteristic to normal intestinal epithelium. Organoids were cultured for 4 days in hanging drops and total cellular RNA or total cellular proteins were isolated from eight drops. Also the total cellular proteins were isolated from organoids cultured for 4 days in Matrigel to compare the protein expression in organoids cultured in both type of models. The results of two separated experiments are shown in both panels (exp. 1, exp. 2). **a** RT-PCR with specific primers revealed in “hanging” organoids the expression of chicken *villin* (marker of mature enterocytes), chicken *sox9* (marker of enterocytes progenitors and intestinal stem cells) and chicken *cdxA* and *cdxB* homeobox genes (caudal family transcription factors expressed both in progenitors and in mature enterocytes). Neg.con. means negative control of PCR reaction, which contained all PCR component with water instead of template cDNA. **b** Immunoblotting with specific antibodies confirming presence of proteins characteristic for: stem cells (Lgr5), proliferating cells (Pcna), progenitors and stem cells (Sox9), mature enterocytes (Sucrase-isomaltase, Villin,) and mesenchymal cells/myofibroblasts (α-SMA), but it should be noted that in “hanging” organoids the expression of α-SMA was minimal

We performed RT-PCR and immunoblot analysis to check the expression levels of markers typical for normal intestinal epithelium in “hanging drop” organoids. We detected the expression (transcripts and protein) of Villin (an enterocyte marker) and Sox9 (a stem/progenitor population marker) (Fig. 3a, b). Furthermore, immunoblotting reveled expression of: Sucrase-isomaltase (an enterocyte differentiation marker), Pcna (a proliferating cell marker) and Lgr5 (a stem cell marker). The expression pattern of specific markers was similar in both types of organoids–hanging drop and insert cultures (Fig. 3b), (Pierzchalska et al. 2012). The presence of Lgr5 stem cells was also previously demonstrated in mouse organoids (cultured in Matrigel) within region called crypt-like domain (Sato et al. 2009, 2011). In mouse intestinal epithelium Lgr5 is expressed in cells called crypt base columnar cells at the bottom of crypts (Barker et al. 2007). These Lgr5 positive stem cells were defined in mammals as self-renewing and multipotent, i.e. developing into all epithelial cell lineages (Rizk and Baker 2012). The presence of Lgr5 positive cells in chicken intestinal organoids indicate that they preserve their proliferating potential in culture.

The expression of caudal family transcription factors (*cdxA* and *cdxB*) was detected in “hanging drop” organoids by RT-PCR, because so far there are no commercially available antibodies that could recognize these proteins in chicken (Fig. 3a). In chicken embryo and also in chickens both *cdxA* and *cdxB* homeobox genes are expressed both in progenitors, as well as in mature enterocytes. The role of these genes in chicken intestine is not entirely clear, but they may be involved in the post-hatch intestinal maturation (Geyra et al. 2002).

Interestingly, myofibroblasts, usually present in the chicken intestinal embryo organoids embedded in extracellular matrix, were very rarely spotted in hanging drop culture (Fig. 1c vs. f). This finding was confirmed by immunoblotting analysis. In the hanging drop organoids α-SMA expression was on the verge of detection limit. On the contrary, in protein lysates isolated from organoids cultured in Matrigel it was evidently identified (Fig. 3b), as well as in protein lysates from the cell and tissue fraction freshly isolated from embryonic intestines in the same way as in case of isolates we typically used for culture establishment (Fig. 6b). In this regard, the organoids in hanging drop culture resemble the organoids originated from adult chicken intestine formed in Matrigel layer, which contain much less myofibroblasts as compared to embryonic organoids (Pierzchalska et al. 2016). The presence of fibroblasts is generally considered beneficial, as they produce various factors that are believed to support stemness (i.e. WNT3a, PGE_2_, angiopoietin-like protein 2) (Lahar et al. 2011; Horiguchi et al. 2017). On the other hand, a situation when two types of cells (myofibroblasts and epithelial cells) are present in the culture model could be a source of misinterpretation, when various tests are performed.

**Fig. 4:**
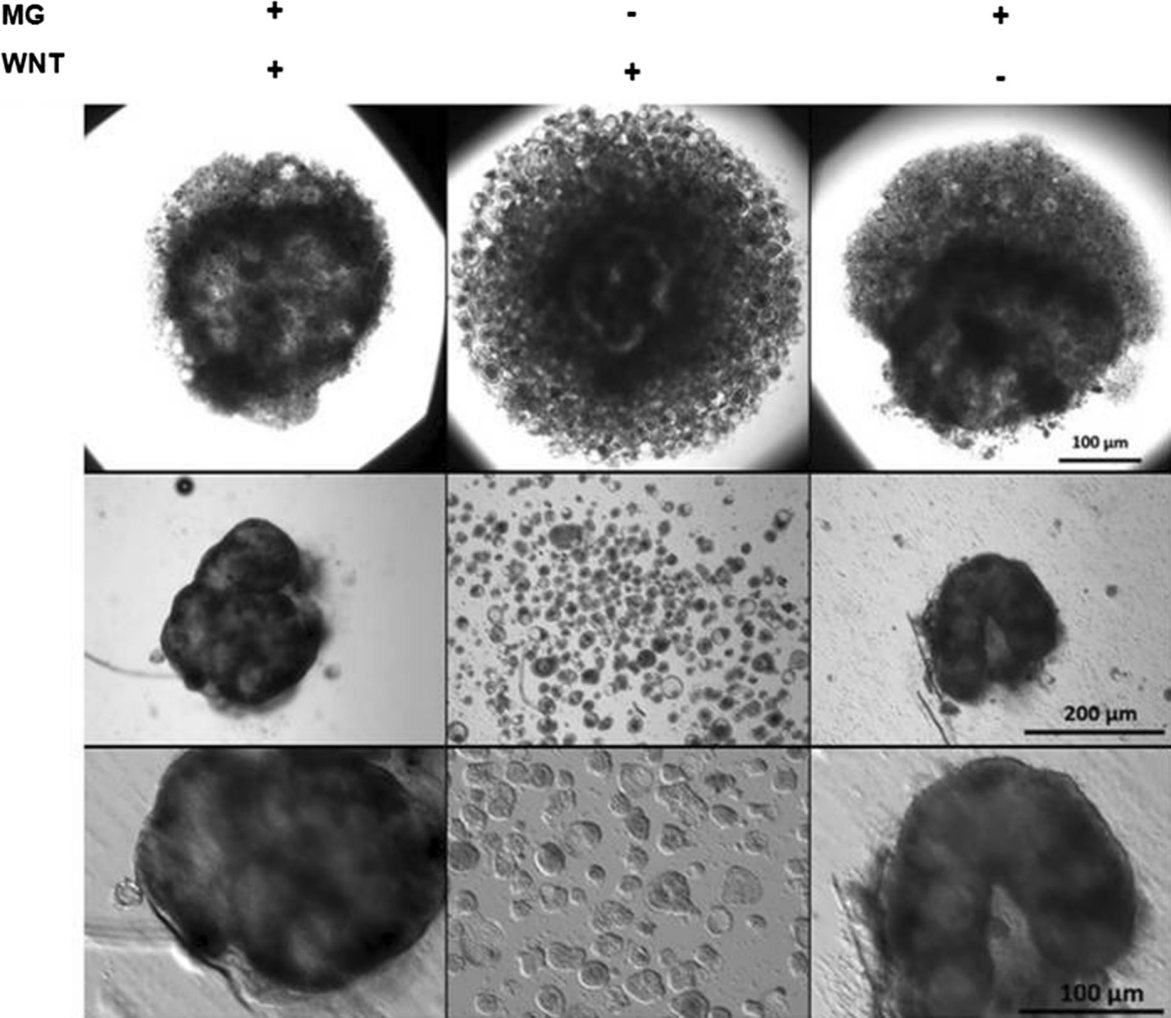
The morphology of hanging drop cultures in the absence or presence of Matrigel and WNT3a in the medium. The epithelial organoids were cultured for 4 days in hanging drop in medium containing Matrigel (5%) and WNT3a (10 ng/ml, left column), in medium with WNT3a but without Matrigel (middle column) or in medium with 5% Matrigel but without WNT3a (right column). Cultures were observed in hanging drop plate with inverted microscope with bright field optics (low magnification-upper column) or transferred into 96-well cell culture plate and photographed with higher magnification and differential interference optics. Organoids formed in the presence of Matrigel create tightly packed aggregates or “superstructures” with various shapes. In the absence of Matrigel only the separated spheroids are present. They are easily detached one from another by any mechanical manipulation (e.g. by an air blowing or a gently pipetting)

**Fig. 5:**
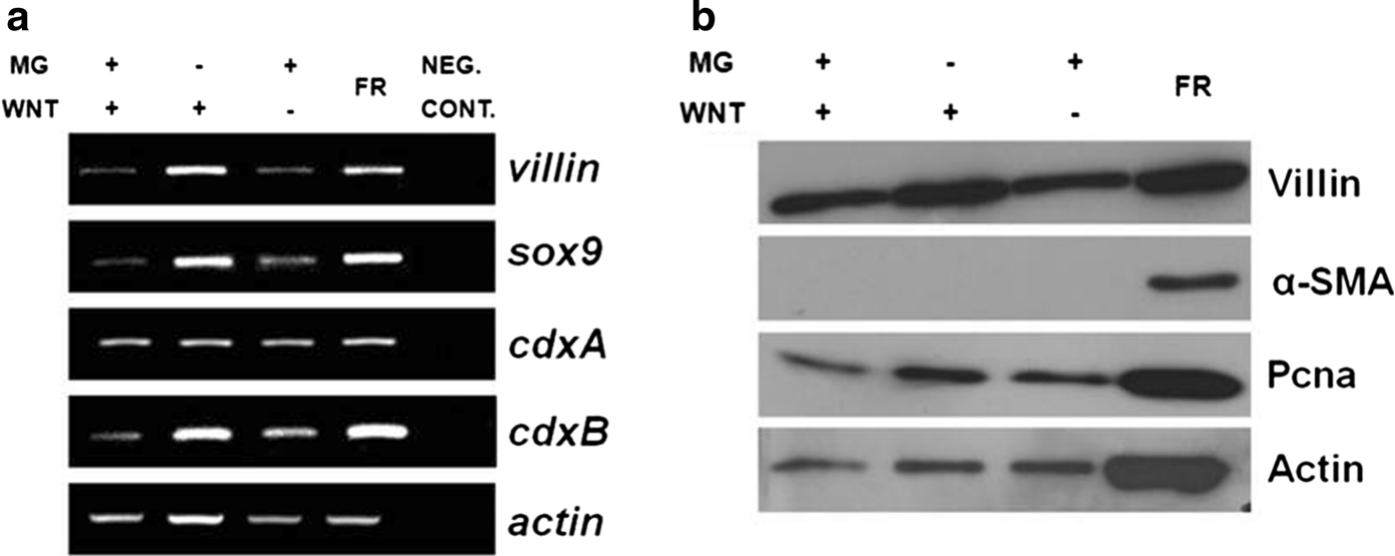
The influence of Matrigel and WNT3a withdrawal on the expression of epithelial progenitors and proliferating cells markers. The total cellular RNA and proteins were isolated from the half of cell and tissue fraction released from chicken embryonic intestines by incubation in EDTA-PBS (FR). The second half of the same fraction was used for culture establishment. The organoids were cultured for 4 days in hanging drops in medium containing Matrigel (5%) and WNT3a (10 ng/ml), in medium with WNT3a but without Matrigel or in medium with 5% Matrigel but without WNT3a and total cellular RNA or total cellular proteins were isolated from 16 or 8 drops, respectively. **a** RT-PCR were performed with specific primers for chicken *villin* (marker of mature enterocytes), chicken *sox9* (marker of enterocytes progenitors and intestinal stem cells) and chicken *cdxA* and *cdxB* homeobox genes (caudal family transcription factors expressed both in progenitors and in mature enterocytes). Neg.con. means negative control of PCR reaction, which contained all PCR component with water instead of template cDNA. **b** The immunoblotting experiments with specific antibodies against Pcna and Villin indicate that in all tested conditions the organoids contain the proliferating cells. Myofibroblasts marker (α-SMA) is present in the initial cell fraction but the protein is nearly not detected after the organoids are formed in “hanging drop” condition

**Fig. 6:**
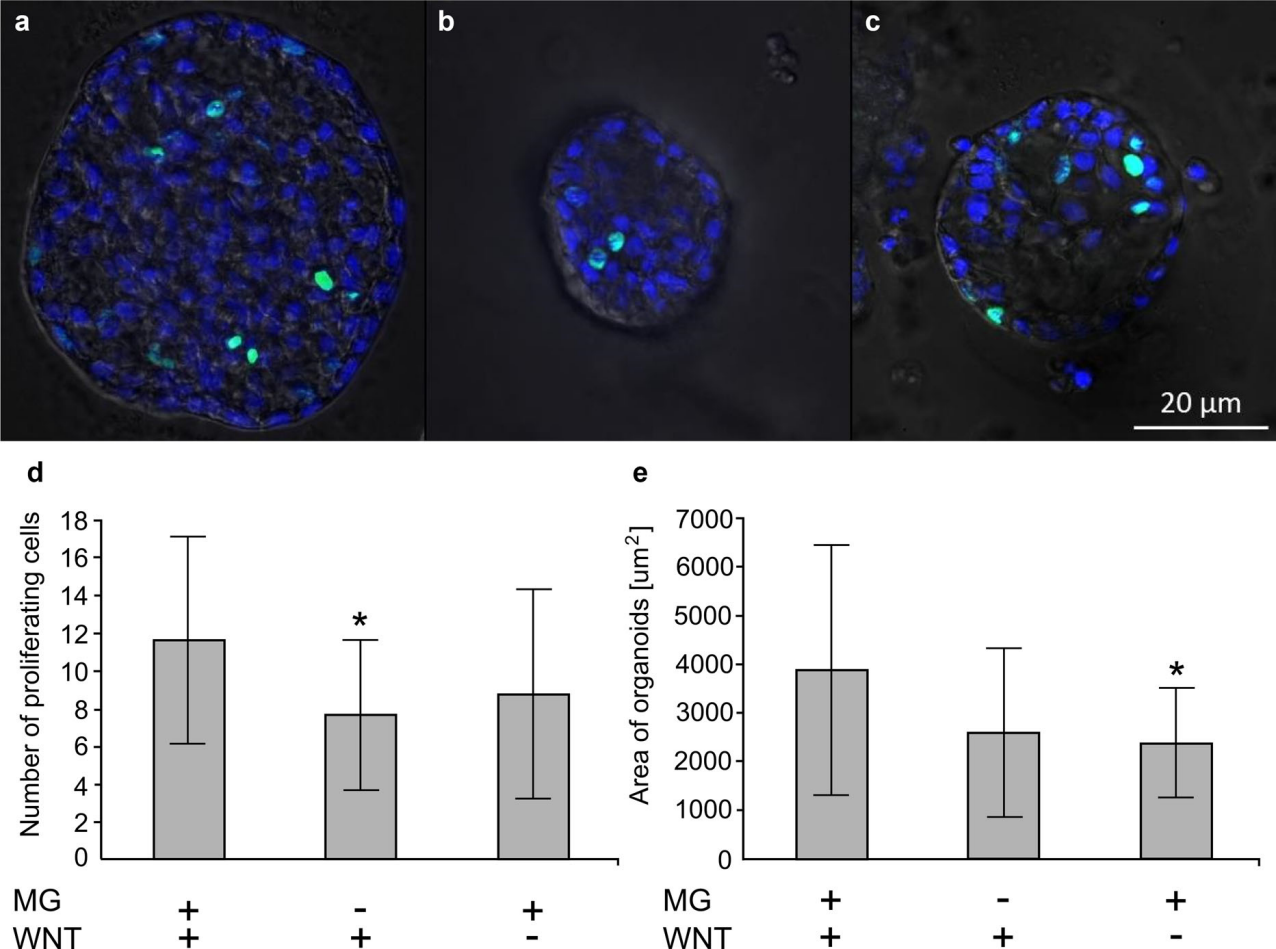
The size of hanging drop organoids and the abundance of proliferating cells in relevance to the presence of Matrigel and WNT3a in the medium. The organoids were cultured for 2 days in hanging drops in medium containing Matrigel (5%) and 10 ng/ml WNT3a (**a**), in medium with WNT3a but without Matrigel (**b**) or in medium with 5% Matrigel but without WNT3a (**c**) and transfer to 96-well plates, fixed and stained with Hoechst 33342 (blue) and with Click-iT^®^ EdU Imaging Kit to detect proliferating cells (green). In each culture condition 15 organoids were analyzed; the area of spheroids projections were measured and the number of proliferating cells was counted in each of them. Graphs show mean values ± SD. The statistical significant differences as tested with contrast analysis (the organoids cultured without Matrigel or WNT3a were compare to standard condition, i.e. medium containing both factors) were marked by asterisk (*p* < 0.05). The organoids formed in medium without Matrigel contained less proliferating cells (**d**) and the organoids were smaller in cultures without WNT3a (**e**). (Color figure online)

We also tested if the medium without any Matrigel addition supports growth of organoids in hanging drops and observed that on day 4 of culture in such conditions they contain only small spherical structures that were not able to aggregate and connect one to another (Fig. 4). This type of culture comprise cells expressing some genes characteristic for the proliferating epithelial cells as was proven by RT-PCR (*cdxA* and *cdxB, sox9*) and the immunoblotting experiments (Pcna) (Fig. 5). Anyway, the typical spheroid formed in such condition is of smaller size, usually lacks the inner empty space and contains less proliferating cells as compared to one grown in media containing Matrigel (Fig. 6).

Hanging drop culture possesses some important advantages over the previously used method of immersing organoids in Matrigel layer. Firstly, in comparison to standard culture in Matrigel, the hanging drop technique allows cost reduction due to smaller quantity of culture media and Matrigel used. In our study, the amount of chicken intestinal epithelial tissue used to obtain one culture in Matrigel was sufficient to prepare eight independent aggregates of spheroids in hanging drop (such situation enables to create more experimental points from the same initial material that is frequently limited). One culture of organoids in Matrigel required about 1 ml of serum free culture medium supplemented with the expensive growth factors, whereas only about 400 µl of culture medium is used for hanging drop preparation of the same scale. Moreover, majority of protocols indicated that 50 µl of Matrigel is necessary to form a culture of organoids in Matrigel, while in hanging drop only 5% of Matrigel addition to the medium is required (e.g. 20 µl per 400 µl of medium, which means about 70% lower expenditure of Matrigel in comparison to the standard protocol). Secondly, the procedure of seeding cells is quicker due to skipping of a Matrigel solidification step. When organoids are cultured in Matrigel, they have to be released from the protein scaffold before most of biochemical analysis are performed. In case of culture in hanging drop, this long step is bypassed as only 5% addition of that basement membrane matrix does not cause medium gelification. For the same reason this type of media has a more defined characteristic and an experimental bias due to the potential heterogeneity in Matrigel’s batches are nearly negligible.

So far, only one published study showed the growth of intestinal organoids in hanging drop, but it employed mouse induced pluripotent stem cell line that formed embryoid bodies and was subsequently differentiated in vitro into a complicated structure containing the mixture of all embryonic germ layers, including mesenchymal cells (Ueda et al. 2010). Our results demonstrate that the culture of organoids derived from intestinal epithelial cells is possible not only in Matrigel matrix, but also in hanging drops. In our opinion it is highly probable that the method presented in this paper could be also successfully employed for the culture of organoids originated from adult intestinal tissue. Our preliminary observations confirm this belief in the case of hens (data not shown) and adult mice (Gawin et al. 2016) intestinal organoids. Generally, our results also suggest that the application of high concentration of ECM proteins and the presence of myofibroblasts are not at all crucial for the formation of intestinal organoids as both of these factors are absent in a hanging drop.
